# The risk of cancer among insulin glargine users in Lithuania: A retrospective population-based study

**DOI:** 10.1515/med-2024-1017

**Published:** 2024-10-15

**Authors:** Justinas Jonusas, Mingailė Drevinskaitė, Donata Linkeviciute-Ulinskiene, Adomas Ladukas, Aušvydas Patašius, Lina Zabulienė, Giedrė Smailytė

**Affiliations:** Laboratory of Cancer Epidemiology, National Cancer Institute, LT-08406, Vilnius, Lithuania; Brachytherapy Department, National Cancer Institute, LT-08406, Vilnius, Lithuania; The Medical Clinic, Västerås Central Hospital, 72189, Västerås, Sweden; Department of Public Health, Institute of Health Sciences, Faculty of Medicine, Vilnius University, LT-03101 Vilnius, Lithuania; Institute of Clinical Medicine, Faculty of Medicine, Vilnius University, 03101, Vilnius, Lithuania

## Abstract

**Objectives:**

The aim of this study was to determine the association between insulin glargine usage and the potential increase in cancer risk among the Lithuanian population diagnosed with type 2 diabetes mellitus (T2DM).

**Methods:**

A retrospective cohort study was conducted. The cohort of insulin users was established by identifying all male and female patients diagnosed with T2DM, as recorded in the National Health Insurance Fund database between 1 January 2000 and 31 December 2012. The risk of cancer among insulin glargine users was compared with the risk in non-glargine insulin users. Cox proportional hazard models were used to estimate hazard ratios (HR) and their 95% confidence intervals (CI).

**Results:**

The overall cancer risk for all sites combined showed no significant difference (HR 0.84, 95% CI 0.67–1.05). Although a general decrease in the risk of cancers was observed at most sites for glargine users, the use of insulin glargine was associated with a non-significant increase in the risk of mouth and pharynx, stomach, non-melanoma skin, breast, cervical, ovarian, and central nervous system cancers. There was a tendency for a lower risk of colon, rectum, rectosigmoid, and anus cancer among glargine users (HR 0.45, 95% CI 0.18–1.12, *p* = 0.09).

**Conclusions:**

Our research contributes to the growing body of evidence showing that insulin glargine is not associated with an increased risk of all cancers or specific types of cancer.

## 1 Introduction

Over the past 30 years, the prevalence of diabetes mellitus (DM) increased from 4.3 (95% CI 2.4–7.0) to 9.0% (95% CI 7.2–11.1), making it one of the most common diseases worldwide [1]. A recent study reported that more than 90% of patients with DM are diagnosed with type 2 diabetes mellitus (T2DM) [2]. It is estimated that more than 6% of the global population is affected by T2DM, and the number is expected to increase in the coming decade [3]. Therefore, the potential association between DM and cancer could significantly impact the general population in the near future [4].

The umbrella review of existing meta-analyses by Tsilidis et al. showed that there is strong evidence for the significant increase in breast cancer, intrahepatic cholangiocarcinoma, colorectal, and endometrial cancer incidence rates among T2DM patients as well as an increased risk of cancer-related mortality [5]. An increased risk of site-specific cancers was found in a cohort of T2DM patients in Lithuania [6]. A study from China showed that individuals diagnosed with DM at age 51–60 years have a higher incidence and mortality from various site-specific cancers. This indicates that the age at diagnosis of T2DM, together with tobacco control, plays an important role in cancer treatment and could present an opportunity for cancer prevention [7]. Hyperinsulinemia, insulin resistance, obesity, and dyslipidemia play a causal role in linking DM to cancer and can significantly promote mutagenesis and carcinogenesis [8]. Elevated insulin secretion from the pancreas into the portal circulation can increase hepatic growth hormone-mediated synthesis of insulin-like receptors growth factor (IGF-1). Epidemiological studies found an association between high-normal levels of insulin, C-peptide, and IGF-1 with an increased risk of various cancers (such as breast, colon, and prostate) [9–11]. Hyperinsulinemia may promote cancer progression either by directly binding to the insulin receptor or indirectly by increasing blood insulin and IGF-1 levels and triggering insulin and IGF-1 signaling that activates the phosphatidylinositol 3-kinase/Akt/mammalian target of rapamycin and mitogen-activated protein kinase pathways, which are crucial for cancer cell proliferation, survival, mobility, and drug resistance [12]. Additionally, in T2DM, metabolic dysfunction triggers a persistent low-grade chronic inflammation marked by an increased release of pro-inflammatory interleukins (ILs), particularly IL-6 and TNF-α, other cytokines (resistin), free fatty acids, C-reactive protein, and other markers of chronic inflammation. Hyperglycaemia and dyslipidemia can promote reactive oxygen species (ROS) production, damaging proteins and DNA with high levels of TNF-α and NF-kB, leading to the proliferation of malignant cells [10,13]. Panigrahi et al.’s investigation showed that diabetes increases DNA damage in breast cancer cells and reduces their ability to repair DNA damage and strand break recovery due to elevated ROS levels [14]. All these factors may increase cancer risk and create an environment conducive to cancer progression.

A discussion on the insulin–cancer relationship includes four consecutive studies arguing that insulin, in particular insulin glargine, used for DM treatment, is associated with an increased risk of cancer [15–18]. A study by Colhoun et al. showed that the incidence of breast cancer was significantly higher in glargine-only users compared to non-glargine users (hazard ratio [HR] 3.65, 95% confidence intervals [CI] 1.05–12.68) [15]. Additionally, a cohort study of more than 127,000 patients with DM from Germany showed a dose–response relationship between glargine use and cancer risk compared to human insulin users [17]. Moreover, a study by Jonasson et al. showed that glargine use was only associated with a higher incidence rate of breast cancer compared to other types of insulin (HR 1.97, 95% CI 1.30–3.00) [18]. Currie et al. showed that all insulin-based therapies were associated with a higher risk of cancer compared to metformin monotherapy (HR 1.42, 95% CI 1.27–1.60). However, there was no significant association between cancer risk and glargine-only use [16].

On the other hand, these studies on insulin use and cancer risk have been criticized due to design flaws [19,20]. Therefore, the uncertainty persists, prompting the scientific community to investigate further. Accordingly, this study aimed to determine whether insulin glargine use is associated with the increase in cancer incidence rate among the Lithuanian population diagnosed with T2DM.

## 2 Materials and methods

### 2.1 Dataset

We used the National Health Insurance Fund (NHIF) database to identify DM patients. This database contains demographic data and entries on primary and secondary healthcare services, emergency and hospital admissions, and reimbursed medication prescriptions.

Cancer cases were identified by record linkage with the Lithuanian Cancer Registry, a nationwide population-based cancer registry that contains personal and demographic information, as well as information on the diagnosis of all people diagnosed with cancer in Lithuania since 1978.

### 2.2 Study design and population

A retrospective cohort study was conducted to examine the relationship between insulin and site-specific cancer risk. The cohort of insulin users was established by identifying all male and female patients who had a first recorded diagnosis of T2DM (International Classification of Diseases Australian modification, ICD-10-AM code E11) in the NHIF database from 1 January 2000 until 31 December 2012. To reduce the possibility of incorrect T2DM classification, patients aged 40 years or older were only included. Furthermore, to ensure that the study cohort included only non-incident insulin users, individuals had to have at least five insulin prescriptions to be included in the study.

**Figure 1:**
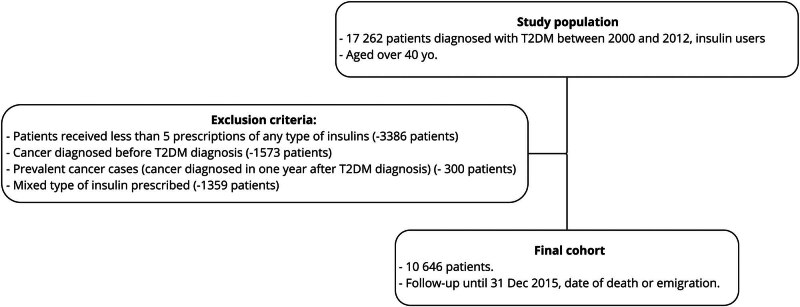
Study flowchart.

To calculate the cancer incidence rate within the cohorts, T2DM records were connected to the Lithuanian National Cancer Registry using a personal identification number assigned to every Lithuanian citizen, using data until 31 December 2015. Participants with a primary cancer diagnosis before receiving insulin (1,573 individuals) or within 1 year after their initial insulin prescription (300 individuals) were excluded (Figure 1).

The remaining cohort included insulin users who were followed for only 1 year after their initial insulin prescription. The cohort’s conclusion date was defined as either the date of death, emigration, or 31 December 2015, whichever occurred first.

### 2.3 Exposure

The different types of insulin prescribed for T2DM were classified into three categories according to the ATC code: insulin glargine (category 1), other insulin analogs (category 2), and human insulin (category 3). Therefore, we have classified patients into two groups: group 1 – insulin glargine-only users (insulin category 1) and group 2 – non-glargine insulin users (insulin categories 2 and 3).

### 2.4 Statistical analysis

Cox proportional hazard models were used to estimate HRs and their 95% CIs to compare the cancer risk of diabetic patients by insulin exposure (glargine vs non-glargine insulin users). In addition, multivariate-adjusted Cox proportional hazards model, including age and gender, were conducted to estimate the effect of insulin type on cancer risk.

All statistical analyses were performed using STATA 15 statistical software (StataCorp. 2020. Stata Statistical Software: Release 15.1. College Station, TX, USA).


**Ethical approval:** All procedures involving human participants were in accordance with the ethical standards of the institutional and/or national research committee and with the 1964 Helsinki Declaration and its later amendments or comparable ethical standards. The study protocol was approved by the Vilnius Regional Biomedical Research Ethics Committee (No. 158200-17-913-423).
**Patient and public involvement statement:** Patients or the public were not involved in our research’s design, conduct, reporting, or dissemination plans.

## 3 Results

Overall, 10,646 individuals (6,573 women and 4,073 men) were included in the study, with 67665.54 person-years of observation. Eight hundred ninety-four patients were treated with non-glargine insulin, while 9,752 individuals used insulin glargine. Insulin glargine users were younger than patients on other insulin types (58.65 vs 65.11 years, *p* < 0.05). Table 1 presents the detailed characteristics of the study population.

**Table 1:** Characteristics of the study population

	All participants	Glargine users	Non-glargine users
T2DM patients, *N* (%)	10,646 (100)	894 (8.40)	9,752 (91.60)
Gender			
Male, *N* (%)	6,573 (61.74)	527 (58.95)	6,046 (62.00)
Female, *N* (%)	4,073 (38.26)	367 (41.05)	3,706 (38.00)
Mean follow-up time, years (SD)	6.36 (2.85)	7.51 (2.48)	6.25 (2.86)
Mean age at the start of follow-up, years (SD)	64.57 (10.52)	58.65 (9.88)	65.11 (10.41)

*N*, number of patients; SD, standard deviation; T2DM, type 2 diabetes mellitus.

The risk of malignancies was assessed by comparing insulin glargine users with T2DM patients treated with other types of insulin (Table 2). There was no significant difference in risk for all cancer sites combined (HR 0.84, 95% CI 0.67–1.05). While there was a general decrease in cancer risk at most sites for glargine users, the use of insulin glargine was associated with a non-significantly increase in the risk of cancers of mouth and pharynx, stomach, non-melanoma skin, breast, cervical, ovarian, and central nervous system. Notably, there was a tendency for a reduced risk of colon, rectum, rectosigmoid, and anal cancer among the glargine users (HR 0.45, 95% CI 0.18–1.12, *p* = 0.09).

**Table 2:** Risk of malignancies in patients using insulin glargine compared to non-glargine users

Primary site	ICD 10-AM	Cancer cases	HR (95% CI)	*p*-value
All sites	C00–C96	82	0.84 (0.67–1.05)*	0.13
Mouth and pharynx	C00–C14	3	2.23 (0.60–8.26)*	0.23
Stomach	C16	5	1.07 (0.43–2.71)*	0.88
Colon, rectum, rectosigmoid, anus	C18–C21	5	0.45 (0.18–1.12)*	0.09
Liver	C22	3	0.79 (0.24–2.58)*	0.69
Pancreas	C25	3	0.68 (0.21–2.21)*	0.52
Trachea	C34	3	0.45 (0.14–1.44)*	0.18
Melanoma	C43	1	1.00 (0.12–8.05)*	0.99
Skin, non-melanoma	C44	12	1.22 (0.66–2.23)*	0.53
Breast	C50	9	1.09 (0.54–2.20)**	0.82
Cervix uteri	C53	2	1.07 (0.24–4.77)**	0.93
Corpus uteri	C54	7	0.87 (0.39–1.94)**	0.74
Ovary	C56	4	1.60 (0.55–4.69)**	0.39
Prostate	C61	14	0.97 (0.55–1.69)**	0.91
Kidney	C64	2	0.37 (0.09–1.52)*	0.17
Urine bladder	C67	1	0.46 (0.062–3.42)*	0.45
Central nervous system	C70-C72	3	1.84 (0.52–6.48)*	0.34
Thyroid	C73	2	0.92 (0.20–4.32)*	0.92
Hodgkin lymphoma, leukaemia, myeloma	C82–C85, C90, C91–C95	2	0.75 (0.23–2.46)	0.64

ICD10-AM – International Classification of Diseases 10, Australian Modification; HR – hazard ratio; CI – confidence interval. *Adjusted for age and gender; **adjusted for age.

## 4 Discussion

The current study shows no significant difference in cancer risk between insulin glargine users and non-glargine users. While some cancer sites showed a higher risk for glargine users and others lower risk compared to non-glargine insulin users, these risk estimates were not statistically significant.

The debate on whether the insulin analog glargine increases the risk of cancer in T2DM patients stems from four epidemiological articles published in Diabetologia in 2009 [15–18]. These articles were criticized for fundamental design flaws, including selection bias, questionable allocation to groups, and unconventional analytical methods [19]. Despite the critiques, these studies were the first efforts in their analysis of the potential effects of insulin glargine on cancer development and have set the way for subsequent research.

According to these publications, a cohort study by Wu et al., including more than 22,000 insulin-treated women with DM, reported 321 cases of breast cancer over a 12-year follow-up [21]. The study was performed using the United Kingdom’s Clinical Practice Research Datalink and showed an increased risk of breast cancer (HR 1.44, 95% CI 1.11–1.85) among insulin glargine users compared to the non-users of insulin glargine. Kostev et al. performed a similar study based on longitudinal data from a large UK database (IMS Disease Analyzer), including demographic information, diagnoses, therapies, and laboratory values, and argued against the likelihood of an actual increase in breast cancer risk with glargine use [22]. In this study, adjusted HRs for breast cancer were 0.92 (95% CI 0.56, 1.50) for LAIA users and 0.98 (95% CI 0.58, 0.65) for insulin glargine users compared to NPH users. A multicentric case–control study performed across 92 institutions in the UK, US, and Canada, including 775 women with DM diagnosed with breast cancer, found no significant risk difference between human insulin users and insulin glargine users (HR 1.29, 95% CI 0.78–2.13) [23]. Similarly, several recent retrospective cohort studies in the US and Europe [24,25], as well as experimental murine model data [26] and a large Taiwanese cohort study of almost 1 million T2DM patients, did not show an increased risk of breast cancer associated with insulin glargine. Our study did not observe an increase in the risk of breast cancer.

In addition, insulin glargine was not associated with a significant increase in the risk of thyroid cancer (HR 1.10, 95% CI 0.89–1.35), which is supported by results of *in vitro* studies showing a non-significant effect of insulin glargine on thyroid cell proliferation and tumor cell migration at low doses [27]. Subsequent cohort studies also showed no significant increase in cancer risk at other sites in T2DM patients taking insulin glargine [28,29]. Our findings are consistent with the existing evidence that insulin glargine does not increase cancer risk in T2DM patients.

Insulin and insulin-like growth factors are known to activate the mitogenic properties of cells by acting on insulin receptors [30]. In addition, insulin phosphorylates the substrates of these insulin receptors, which subsequently promote growth, proliferation, mobility, and migration [31]. Studies have shown that insulin glargine binds to insulin receptors with an affinity similar to human insulin but with a much higher affinity to insulin-like growth factor receptors [32]. This finding is supported by another study, showing that insulin glargine is more effective in activating insulin-like growth factor receptors [33]. Additionally, insulin-like growth factors have been shown to be strong mitogens in the pancreas, kidneys, prostate, breast, and other solid tumors [34,35]. It is, therefore, not surprising that the mitogenic potential of insulin glargine is almost eight times higher than that of human insulin [32]. However, insulin and IGF-1 receptor signaling is influenced by various factors, such as the duration of contact between ligand and receptor and the dissociation rate [36]. Moreover, insulin glargine is reported to be transformed by cleaving at the carboxy terminus of its B-chain, resulting in the formation of two metabolites, the potency of which in activating IGF-1 receptors is significantly reduced after the injection into the circulation or subcutaneously [37]. This could explain our data, which suggests that insulin glargine is not associated with an increased cancer risk.

Since 2011, the American Diabetes Association (ADA), in their Standards of Medical Care [38], included the review by Giovannucci et al. [39] on diabetes and cancer, pointing attention to the problem of diabetes and cancer risk. Therefore, since 2012, the ADA has recommended that patients with diabetes be encouraged to undergo age- and sex-specific appropriate cancer screenings, as recommended by their primary healthcare professional [40]. Later on, recommendations to reduce their modifiable risk factors for cancer (such as obesity, physical inactivity, and smoking) were added in 2024 [41]. ADA and the European Association for the Study of Diabetes (EASD) do not currently recommend against using insulin glargine. Although some observational studies raise concerns over the increased risk of cancer, the overall evidence does not justify a change in clinical practice for the use of insulin glargine. Both organizations stress that the benefits of good glycaemic control with insulin treatment, including insulin glargine, outweigh the potential risks [41,42]. The ADA, EASD, and the American Association of Clinical Endocrinology recommend continued monitoring and research in this area to ensure patient safety. This is due to endogenous hyperinsulinemia being a suggested factor for the link between cancer, obesity, and DM. Consequently, it is crucial to improve our understanding of how insulin therapy affects cancer risk and progression [43].

Although our study is a retrospective cohort study, it was carefully designed to include only patients who received both glargine and non-glargine insulin, with follow-up started 1 year after the first medication prescription. The strengths of our study include the long follow-up period and the comprehensive inclusion of the entire regional population with verified exposure. However, our study has several limitations. First of all, our study investigated the association between insulin glargine use and cancer risk. However, as an observational study, its findings should be interpreted with caution, as observational studies can identify correlations but are not designed to establish causality. Furthermore, the sample size of T2DM patients who were treated with insulin glargine alone was relatively small, and the study duration from 2000 to 2012 may not be sufficient to observe the potential long-term effects of insulin glargine on cancer risk. This limitation may affect our ability to detect cancers that develop later or comprehensively evaluate the long-term risks associated with insulin glargine use. Third, our focus was solely on the insulin analog glargine as the insulin analog detemir was not yet available in the market. In addition, the study also did not account for potential confounding factors such as lifestyle, dietary habits, body mass index, smoking, and comorbidities due to the retrospective study design. Lastly, our dataset lacks information on the doses of insulin glargine used by patients, which could be a significant factor in cancer risk as suggested by Hemkens et al. [17]. Due to this limitation, we could not analyze or discuss association of insulin dosage on cancer risk in our study. Future research should include larger populations, longer follow-up duration, insulin doses used by patients with DM, and analyze confounding factors to improve understanding of potential long-term cancer risks.

## 5 Conclusions

Our research contributes to the growing body of evidence showing that the use of insulin glargine is not associated with an increased risk of either all cancers or specific types of cancer.
